# Data for estimating the U.S. labor wedge

**DOI:** 10.1016/j.dib.2018.04.128

**Published:** 2018-05-24

**Authors:** Lini Zhang

**Affiliations:** Central University of Finance and Economics, China

**Keywords:** Labor wedge, GDP, Business cycle, MRS, MPL

## Abstract

The after-tax labor wedge is defined as the log difference between the MRS and the MPL excluding taxes. This article introduces the data and approach that are used to estimate the U.S. after-tax labor wedge to provide empirical support for the research article entitled "Credit Crunch, Heterogeneity and the Labor Wedge" (Zhang, 2018 (Forthcoming)) [4]. I measure the U.S. after-tax labor wedge and then decompose it into the sum of the gap between the MPL and the real wage (the MPL component) and the gap between the real wage and the MRS (the MRS component). The after-tax labor wedge and its decomposition are measured using quarterly data from 1947Q1 to 2017Q3.

**Specifications Table**

**Table:** 

Subject area	Economics
More specific subject area	Macroeconomics
Type of data	Excel file
How data was acquired	Bureau of Economic Analysis and Business Dynamic Statistics
Data format	Analyzed
Experimental factors	The real variables are calculated using the GDP deflator. The Labor Wedge are detrended using the Hodrick-Prescott filter with a smoothing parameter of 1600.
Data source location	Data on aggregate output and consumption are taken from Table 1.1.5 of the BEA. Labor hour is measured following the data sources in Cociuba, Prescott and Ueberfeldt [2]. Tax wedge is measured using the data source mentioned in Karabarbounis [3]
Data accessibility	All data used in this article is publicly available.

**Value of the Data**

● All data applied in this article is publicly available.
● The article describes the approach to measure and decompose the after-tax labor wedge
● The data and methods allow other researchers to replicate and extend the analysis.

## 1 Data

All data for estimation in this article extend from 1947Q1 to 2017Q3. Output is the Real Gross Domestic Product (billions of chained 2009 dollars) from Table 1.1.6 of the Bureau of Economic Analysis (BEA). Consumption is Personal Consumption Expenditures less durable goods from Table 1.1.5 of the BEA. Capital is the sum of private fixed assets and consumer durable from Fixed Asset Table 1.1 of the BEA. Establishment entry and exit ratio is from the Business Dynamic Statistics. Data on labor hour is constructed by Cociuba et al. [2]. I extended their labor estimation from 2012Q1 to 2017Q3 based on their method and data sources. For the measurement of U.S. taxes, net taxes on production and imports is from Table 1.12 of the BEA. Personal current taxes and contributions for government social insurance are from Table 3.1 of the BEA. Data on the labor share is from the BLS.

## 2 Experimental design, materials, and methods

The labor wedge, which is defined as the discrepancy measured in data between the marginal product of labor (MPL) and the marginal rate of substitution (MRS), is important for understanding the limitation of economic models and guiding economists to design policies that can improve economic efficiency. Over the 2007–2009 financial crisis, the labor wedge excluding taxes has experienced significant increases, which is not well understood in the literature. Thus the related research article [4] develops a heterogeneous entrepreneurial model with credit constraints to explain the movements of the after-tax labor wedge during this period. In this article, I focus on describing the associated data and approach for measuring the U.S. after-tax labor wedge. Besides measuring the labor wedge, I also decompose the labor wedge into a MRS component and a MPL component following Karabarbounis [3] because such decomposition is important for understanding the nature and movements of the labor wedge studied in the related research article.

### 2.1 Definition and derivation of the labor wedge

In Chari et al. [1] and Karabarbounis [3] the after-tax labor wedge $\tau_{l}$ is equal to the log difference between MPL and the MRS:

$$\tau_{l}=\log\left(\mathrm{MPL}\right)\;-\;\log(\mathrm{MRS})$$

where

$$\mathrm{MRS}=\;\frac{u_{2}\left(C,\;1-L\right)}{u_{1}\left(C,\;1-L\right)}\frac{1+T^{c}}{1-T^{n}}$$

and

$$\mathrm{MPL}=(1-\alpha )\upsilon \frac{Y}{L}.$$

Parameters C, L, Y are aggregate consumption, labor and output, respectively; $T^{n}$ is labor tax rate and $T^{c}$ is consumption tax rate; $u\left(C,\;1-L\right)=\;\frac{{{(C}^{\tau }{\left(1-L\right)}^{1-\tau })}^{1-\sigma }}{1-\sigma }$ is the household utility function. Output $Y=z{(K^{\alpha }L^{1-\alpha })}^{\upsilon }$, where z is aggregate productivity, α is the share of output that goes to capital and υ is the production return to scale parameter. When υ<1, it means that the production has decreasing returns to scale.

According to Karabarbounis [3] , the labor wedge can be decomposed as the sum of the gap between the real wage $\tau^{h}$ and the MRS and the gap between MPL and the real wage $\tau^{f}$. That is,

(1) $${\;\tau }_{l}\;=\;\tau^{h}\;+\;\tau^{f}$$

(2) $${\;\tau }^{f}\;=\;\log\left(1-\alpha \right)\;-\;\log(s)$$

(3) $${\;\tau }^{h}\;=\;\log\left(\frac{1}{1-\tau }\right)\;+\;\log\left(s\right)\;+\;\log\left(\frac{1-L}{L}\right)\;+\;\log\left(\frac{Y}{C}\right)\;-\;\log(\frac{1+T^{c}}{1-T^{n}})$$

where $\tau_{l}$ is the labor wedge, $\tau^{h}$ is the gap between MRS and the real wage, $\tau^{f}$ is the gap between MPL and the real wage. Parameter $s_{t}=\frac{w_{t}L_{t}}{y_{t}}$ is the labor share of income in the data, Notice that parameterτis from the utility function.

I measure the labor wedge and its two major components defined in Eqs. (1), (2), (3) using the data described in the Data Section. The real values of aggregate consumption C, output Y and capital K are calculated using the GDP deflator. Additional to the labor wedge, I also estimated the Solow Residual, z, using data on output, capital and hours. To achieve that I detrended the real output and consumption using the Hodrick-Prescott filter with smoothing parameter 1600. Since the Hodrick-Prescott filter is known to distort the fitting trend at the two ends of the data series, the estimation results from 1948Q1 to 2015Q4 are used and shown.

### 2.2 Taxes measurement

Following the method and data sources in Karabarbounis [3], I estimated the consumption taxes $T^{c}$ and labor income taxes $T^{n}$. Consumption tax rate is consumption taxes divided by personal consumption expenditures less consumption taxes paid. Consumption taxes equals net taxes on production and imports data (BEA NIPA Table 1.12). The labor income tax rate is measured as the sum of personal income tax rate and social insurance tax rate. Personal income tax rate is personal current taxes (BEA NIPA Table 3.1) divided by GDP less taxes on production and imports. Social insurance tax rate is contributions for government social insurance (BEA NIPA Table 3.1) divided by measured national income, which equals the product of the labor share and real GDP less net taxes on production and imports. Data on the labor share is from the BLS.

### 2.3 Parameters selection

**Fig. 1:**
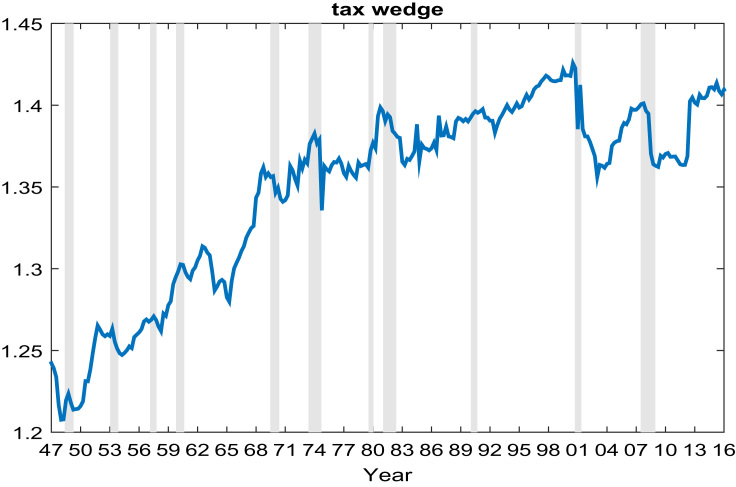
The tax wedge. The tax wedge $\frac{\left(1+T_{c}\right)}{\left(1-T_{n}\right)}$ is the ratio of consumption tax rate T_c_ and labor income tax rate T_n_.

**Fig. 2:**
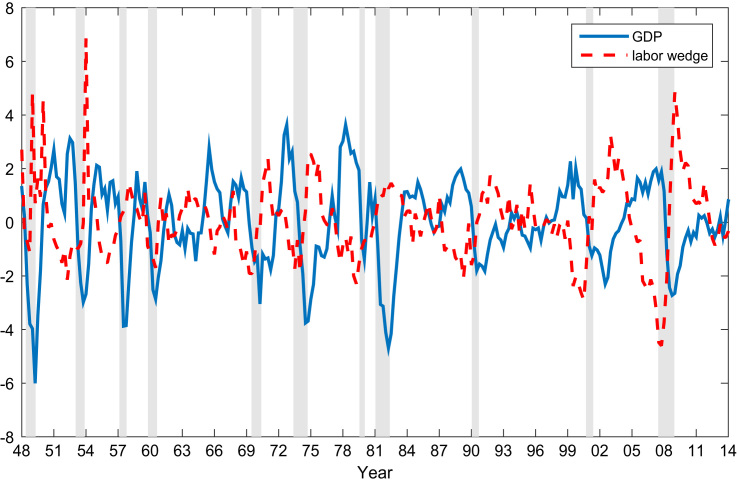
The cyclical variations of real GDP and the labor wedge.

**Fig. 3:**
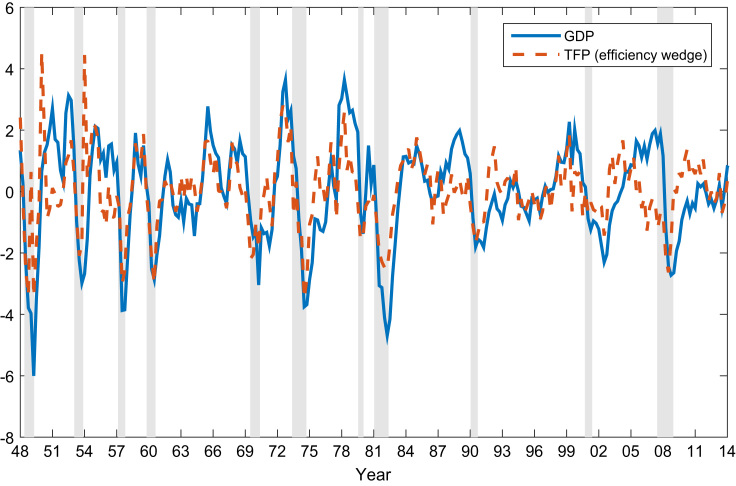
The cyclical variations of real GDP and the TFP (Solow Residual).

**Fig. 4:**
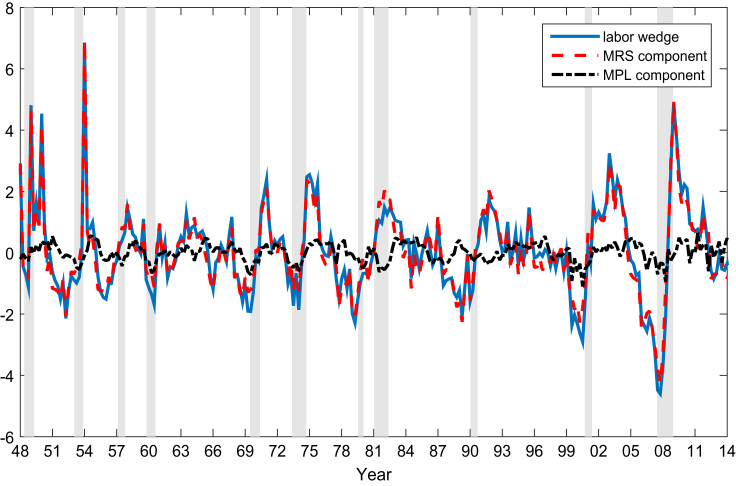
The cyclical variations of the labor wedge, and the MRS and MPL components.

Since Karabarbounis [3] did not disclose his choices forαandτ, I adopted the values from the steady state calibration of my research paper associated with this Data-In-Brief article. Capital share of incomeαequals 0.3, which is consistent with most macroeconomic literature. Parameterτis 0.38 as determined by calibration. Notice thatαandτdoes not change over time, so they would not affect the fluctuations of the labor wedge over the business cycles. Sinceαandτonly affect the trends of labor wedge, their impacts on the measurement of the labor wedge in Fig. 1 of the paper is trivial. Finally, since the labor wedge and its components exhibits trends, the labor wedge shown in figures below is detrended using the Hodrick-Prescott filter with smoothing parameter 1600 (Fig. 2, Fig. 3, Fig. 4).
