# Supplementary material for: Data for estimating the U.S. labor wedge
Source: Data Brief. 2018 May 24;20:562–7. doi: 10.1016/j.dib.2018.04.128 (PMC6126211; doi:10.1016/j.dib.2018.04.128)
Supplement: Supplementary file 1 — Supplementary material [file mmc1.pdf]

Author Declaration

I wish to confirm to the Editor that all data used in this article is publicly available and there is no potential conflicts of interest.
